# Comparing Intensive and Conventional Therapy: A Meta-Analysis of Postoperative Physical Outcomes After Total Knee Replacement

**DOI:** 10.7759/cureus.88501

**Published:** 2025-07-22

**Authors:** Mohamed Zahed, Alzahraa Faris Alesawy, Ziad Samir Zahed, Rahafat Samir, Mahmoud Eleisawy

**Affiliations:** 1 Orthopedics, John Radcliffe Hospital, Oxford University Hospitals NHS Foundation Trust, Oxford, GBR; 2 Clinical Microbiology and Immunology, Faculty of Medicine, Benha University, Benha, EGY; 3 Ophthalmology, Faculty of Medicine, Benha University, Benha, EGY; 4 Ophthalmology, Benha University Hospitals, Benha University, Benha, EGY

**Keywords:** meta-analysis, osteoarthritis, post-operative outcomes, systematic review, total knee arthroplasty

## Abstract

Osteoarthritis (OA) is a common degenerative joint disease causing cartilage damage, bone erosion, and chronic pain, often leading to disability. Total knee arthroplasty (TKA) is frequently performed to relieve OA symptoms. Conventional therapy training (CTT) is the standard intervention, whether preoperative or postoperative. We assume that intensive therapy training (ITT) may have higher effects in some aspects. The study aims to evaluate the impact of postoperative CTT versus ITT on various physical measures and questionnaires over different follow-up periods.

Our systematic review and meta-analysis followed the Preferred Reporting Items for Systematic Reviews and Meta-Analyses (PRISMA) and Cochrane guidelines, searching until June 2024. We assessed the risk of bias using the Cochrane Risk of Bias 2 (ROB 2) tool. Data were analyzed using Review Manager 5.4 (Cochrane Collaboration, London, UK), with mean differences (MD) and 95% confidence intervals (CI), and heterogeneity was assessed via P-value and I^2 ^tests.

The study consisted of 1087 patients. In the first month of follow-up, ITT did not significantly reduce pain on the visual analog scale (VAS) compared to CTT, with similar results at three and 12 months (overall MD = -0.38, 95% CI = -1.56 to 0.8, P = 0.53). For Western Ontario and McMaster Universities Osteoarthritis Index (WOMAC) scores, ITT showed significant improvement within the first week and at one month (MD = -14.60 and MD = -3.11, respectively), but not at later follow-ups. In range of motion (ROM) flexion, ITT significantly improved outcomes in the first week (MD = 8.60, P = 0.001), but showed no significant differences at one and three months. No other outcomes showed any significant difference, and both results in ITT and CTT were similar.

In TKA rehabilitation, ITT provides early benefits, particularly in improving ROM flexion and WOMAC scores during the initial postoperative week. However, ITT does not show significant advantages over CTT in terms of walking distance, quadriceps strength, ROM extension, or pain reduction throughout various follow-up periods. While ITT offers slight early gains, it does not present long-term benefits over CTT. Incorporating preoperative training into the postoperative regimen may be beneficial. We recommend that high-intensity exercises may not be necessary, as they yield similar results to conventional methods. However, further research is needed to explore both early and long-term outcomes that are not fully addressed in current studies.

## Introduction and background

Osteoarthritis (OA) is a progressive degenerative joint disease that damages the cartilage and causes bone erosion. Misrepresentation of OA is associated with chronic pain that causes disability [1]. OA is considered the most frequent joint disorder worldwide [1]. Over 700,000 total knee arthroplasties (TKAs) are conducted in the US to relieve the pain caused by OA, and this number is expected to rise, reaching 3.5 million cases annually by the year 2030 [2]. The number of people with OA is increasing due to an aging population and the growing prevalence of risk factors such as obesity and reduced physical activity, as highlighted by the methodology used in the Global Burden of Disease study [3].

TKA is an operative option for OA treatment; it is indicated for disability, pain, and dysfunction [4-6]. TKA has demonstrated good clinical outcomes, improving not only the functional impairment but also the daily activities [7]. On the other hand, the occurrence of continuous mild and infrequent pain has been reported after TKA, and 15% of patients experience severe pain three to four years after surgery [8]. Various factors, such as genetic, psychological, and other clinical factors, result in continuous pain after TKA [9,10]. TKA procedures employ one of three fixation techniques: cemented, cementless, or hybrid; all showed similar results regardless of the technique or the component used [11].

The physiotherapy programs for TKA can be conducted in both the preoperative and postoperative periods, as they can be extended over time, whether for rehabilitation or long-term follow-up after surgery. The preoperative training for patients with end-stage OA boosts the preliminary outcomes and improves the patient’s satisfaction after TKA [12]. Postoperative exercise focuses on pain management, reducing swelling, and improving the knee's range of motion [13]. Since the most significant loss of strength and functional performance occurs during the first month after a TKA, low-intensity exercises are necessary to alleviate symptoms and promote recovery speed [5,14]. Another hypothesis is that the high-intensity training postoperatively would be associated with better results regarding the outcomes [15].

Rehabilitation programs are not specific or standardized. The current strategies focus on low-intensity exercise [16,17]. However, recent studies suggest that higher intensity types of training may result in better results and be associated with functional strengthening and function of the knee while maintaining safety [14]. In this study, we aim to compare the safety and effectiveness of intensive therapy training (ITT) with conventional therapy training (CTT) on the outcomes of TKA.

## Review

Methods

Our systematic review and meta-analysis were performed according to the Preferred Reporting Items for Systematic Reviews and Meta-Analyses (PRISMA) statement and the guidelines of the Cochrane Handbook for systematic reviews [18,19].

Searching Databases and Keywords

Two authors searched ClinicalTrials.gov and five databases (PubMed, Cochrane Library, Web of Science, Scopus, and Embase) until June 2024 using the search terms: (("heavy" OR "progressive" OR "maximal" OR "explosive" OR "resistance" OR "high-intensity" OR "intensified" OR "strengthening" OR "weight lifting" OR "weight bearing" OR "concentric" OR "eccentric" OR "endurance" OR "elastic tube" OR "pulleys") AND ("arthroplasty" OR "replacement" OR "surgery" OR "operation") AND (knee) AND ("training" OR "exercise" OR "rehabilitation") AND ("randomized" OR "randomly" OR "randomised" OR "random")).

The search was performed without any time or language restrictions and was supplemented with a manual search of the references in the included studies.

Eligibility Criteria and Study Selection

The PICOS (population, intervention, comparison, outcome, and study design) framework was employed to select search terms, develop a search strategy, and establish inclusion criteria [20]. The PICOS parameters were defined as follows: population (patients with recent knee arthroplasty); intervention (intensive physical therapy, postoperative only); comparison (conventional physical therapy, which is applied postoperatively); outcome (clinical outcomes and questionnaires); and study design (randomized clinical trials).

Exclusion criteria included observational studies (cohort, case series, and case reports), reviews, animal studies, qualitative studies, and single-arm studies.

Risk of Bias Assessment

This review ensured data quality by having two researchers independently extract information from the included trials. To address any disagreements, other authors provided supervision and helped reach a consensus. Furthermore, the risk of bias in the studies was assessed using appropriate tools. Randomized controlled trials (RCTs) were evaluated using the Cochrane Risk of Bias 2 (ROB 2) tool, which examines five key areas [21].

Data Extraction

Two authors independently used Excel spreadsheets (Microsoft Corporation, Redmond, WA) to extract data, including (1) summary data (study time and sites, study design, protocol number, total number of patients, ITT details, CTT details, follow-up duration, and primary outcome); (2) baseline patient data (study groups, age, gender, BMI, height, and knee arthroplasty laterality and location); and (3) clinical outcomes. Clinical outcomes were classified into two categories: physical measures (six or 10-minute walk test, quadriceps strength, range of motion (ROM) extension, ROM flexion, stair test in seconds, and timed up and go (TUG) test) and questionnaires (Western Ontario and McMaster Universities Osteoarthritis Index (WOMAC) and visual analog scale (VAS)).

Data Analysis

Review Manager version 5.4 software (Cochrane Collaboration, London, UK) was used to carry out the analytic part. The results of the continuous outcomes were compiled into a mean difference (MD) coupled with a 95% confidence interval (CI). The data were deemed statistically significant if the P-value was below 0.05. The χ² and I-square (I²) tests were used to assess heterogeneity. Heterogeneity was deemed present when the χ2 P-value was less than 0.1 or when the I² value exceeded 50%. The random-effect model was chosen for the situations in which heterogeneity was identified, whereas the fixed-effect model was utilized for all the remaining scenarios. The out method was tested if the random effect did not solved the heterogeneity.

Results

Literature Search and Study Selection

**Figure 1:**
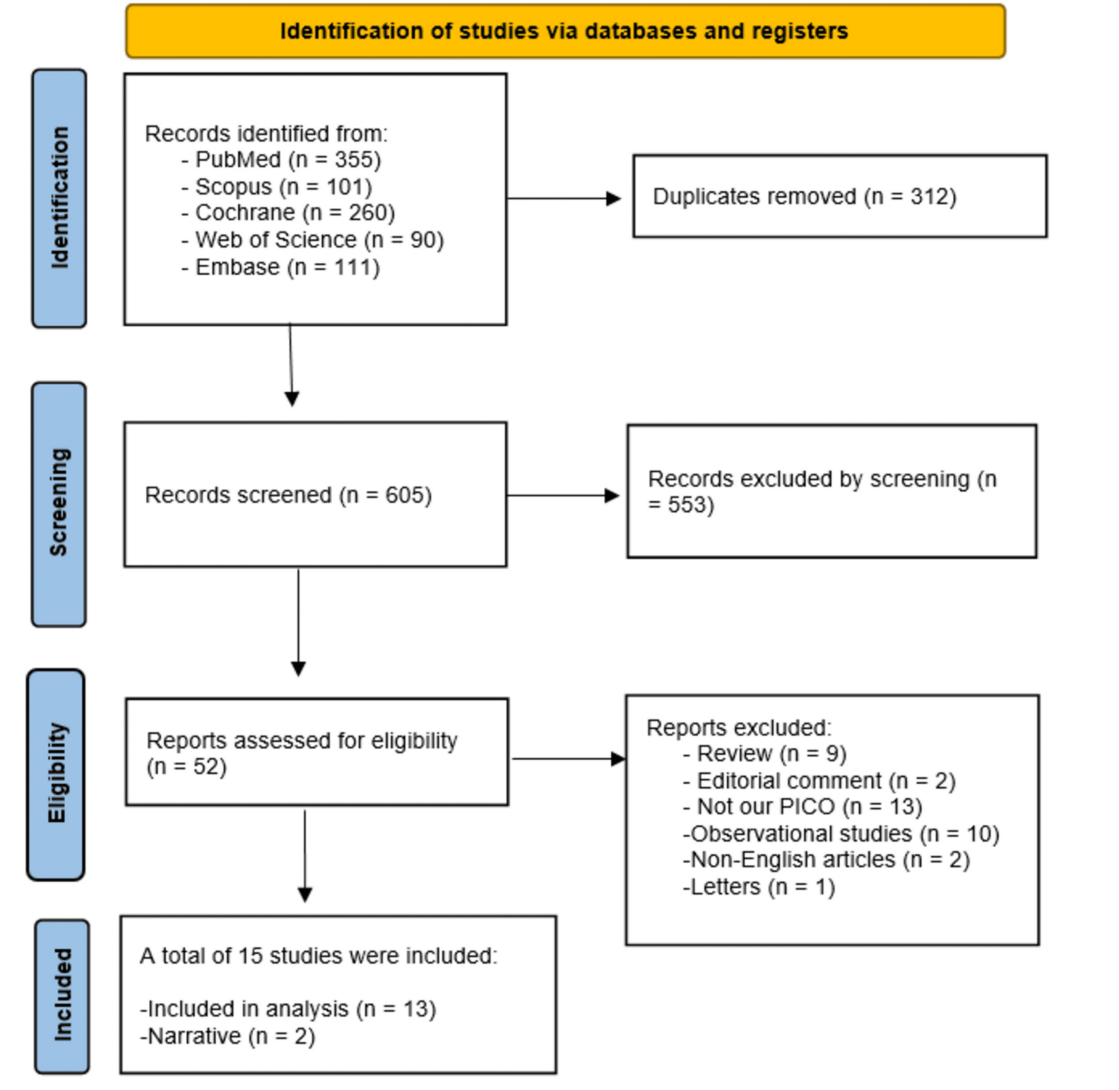
PRISMA flow diagram. PRISMA: Preferred Reporting Items for Systematic Reviews and Meta-Analyses.

According to our search strategy, we found a total of 917 articles, and after removing the duplicates, a total of 605 studies proceeded for the title and abstract screening. After title and abstract screening, followed by full-text screening, a total of 15 studies [22-36] were suitable for our inclusion criteria. Full details are shown in Figure 1.

Study Characteristics

We included 15 studies. All of these studies were RCTs. The total population was 1087 patients. These studies were conducted in different countries and centers, such as the USA, Korea, Taiwan, France, Japan, Norway, China, Denmark, the Netherlands, the United Kingdom, and Spain. The studies were conducted from 2006 to 2024. The follow-up duration ranged from three days, which was mentioned in only one study, up to one year, which was evaluated in five of the included studies. We observed that most of the included population was older, with more than 60 years of age. The predominant gender was female, as OA is higher in incidence among females, except for Lee et al. [28], which only included males with no females included. The majority of the patients had unilateral knee arthroplasty, except for one study. Full details are described in Tables 1, 2.

**Table 1:** Summary of the included studies. ROM: range of motion; WOMAC: Western Ontario and McMaster Universities Osteoarthritis Index; 6MWT: six-minute walk test; RCT: randomized controlled trial; NR: not reported; RM: repetition maximum; PST: progressive strength training.

Study ID	Study time and sites	Design and phases	Protocol number	Total number of patients	Intensive therapy details	Conventional therapy details	Follow-up duration (months)	Primary outcome
Bade et al. (2017) [22]	USA, 2011- 2014	RCT	NCT01537328	162	TYPE: postoperative rehabilitation program. TIME: starting from 3 to 5 days postoperative. DURATION: 11 weeks. NUMBER OF SESSIONS: 26 sessions. TYPES OF EXERCISES: warm up, strengthening exercises included in the progressive rehabilitation program target the following muscles: ankle plantar flexion/extension, quadriceps, hamstrings, hip abductor, knee flexor/extensor. Based on 2 sets/8 repetitions, balance exercises, agility exercises, and weight-bearing exercises.	TYPE: as the intervention group. TIME: as the intervention group. DURATION: as the intervention group. NUMBER OF SESSIONS: as the intervention group. TYPES OF EXERCISES: low-intensity exercises (isometric exercises, ROM exercises), slower transition to weight-bearing exercises, restriction of daily activities.	12	The stair-climbing test
Husby et al. (2018) [25]	Norway, 2013-2014	RCT	NCT01877733	41	TYPE: postoperative rehabilitation program. DURATION: 8 weeks. NUMBER OF SESSIONS: 24 sessions. TYPES OF EXERCISES: warm up, walking, strength training (leg press, knee extension) based on 4 sets, 5 repetitions, with load 90% of 1RM (resting period of 1-2 minutes when reaching 6RM, leg press increased by 5k, knee extension by 1k). PROGRAM INTENSITY: corresponds to a load of 90% 1RM.	TYPE: postoperative rehabilitation program. DURATION: 8 weeks. TYPE OF EXERCISES: low-intensity exercises (non-weight-bearing exercises).	12	1 repetition maximum leg press
Jørgensen et al. (2017) [27]	Denmark, 2011-2013	RCT	NCT01345825	55	TYPE: postoperative rehabilitation program, besides a home training program. TIME: 1 week after surgery. DURATION: 2 weeks. NUMBER OF SESSIONS:16. TYPES OF EXERCISE: warm-up strengthening exercises (leg press, knee extension, knee flexion) based on progression from 12 repetitions maximum to 8 repetitions maximum from 2 sets to 4 sets, rest between exercises of 2-3 minutes.	TYPE: postoperative home training program. TYPE OF EXERCISES: a group of exercises aiming to increase blood and lymph circulation in the limb, and increase the knee range of motion. Low-intensity strength exercises were performed 6 weeks postoperatively, 3 sets/10 repetitions	12	1. Leg extension power. 2. Knee Injury and Osteoarthritis Outcome Score
Lenssen et al. (2006) [30]	Netherlands, 2004	RCT	NR	43	TYPE: postoperative rehabilitation program. DURATION: 4 days. NUMBER OF SESSIONS: 7 sessions, 2 sessions per day (40-minute session duration). TYPES OF EXERCISES: active and passive mobilization of the knee, strengthening of the quadriceps muscle, and functional exercises, including transfers from a supine position to sitting.	TYPE: as the intervention group. DURATION: 4 days. NUMBER OF SESSIONS: 4 sessions, 1 session per day (20-minute session duration). TYPE OF THERAPY: as the intervention group.	3	Range of motion
Lowe et al. (2012) [32]	United Kingdom, 2007-2009	RCT	ISRCTN07624314	107	TYPE: postoperative rehabilitation program, plus the conventional usual physiotherapy treatment. TIME: within 2 weeks and between 6 and 8 weeks. NUMBER OF SESSIONS: 2 sessions. TYPE OF EXERCISE: (weight shift, partial knee band, standing knee flexion, knee raise, step over, quadriceps stretching) based on 5 repetitions of stretching, each for 5 seconds, repetitions of all other exercises twice daily for at least three months. PROGRAM INTENSITY: repetition rate, frequency progressions left to physiotherapist.	TYPE: postoperative conventional usual physiotherapy treatment. TYPES OF EXERCISES: static quadriceps contractions, inner range quadriceps contractions, and knee flexion. Standing exercises: knee extension (with hamstring stretch) and sit-to-stand.	12	Oxford Knee Score
Nakamura et al. (2020) [33]	Japan, 2015-2016	RCT	NR	49	TYPE: postoperative rehabilitation program (physical and high-intensity balance). TIME: from day 1 to 4 postoperatively. DURATION: 21 days. NUMBER OF SESSIONS: 12-14 sessions. PROGRAM EXERCISES: Physical (ROM, basic motion exercises (walking)), balance exercises (stepping), and muscle strength exercises (squatting, hip abductors, and rotator cuffs), balance (standing, stepping, and other exercises). PROGRAM INTENSITY: training loads were adjusted on a seat-to-seat basis to reflect relative loads.	TYPE: postoperative rehabilitation program (physical only). TIME: day 1 postoperative. DURATION: 21 days. NUMBER OF SESSIONS: 12-14 sessions. PROGRAM EXERCISES: ROM, basic motion exercises (walking), balance exercises (stepping), and muscle strength exercises (squatting, hip abductors, and rotator cuffs).	1	1. Balance ability. 2. The standing position control ability
Núñez-Cortés et al. (2024) [34]	Spain, 2022	RCT	NCT05444400	40	TYPE: postoperative rehabilitation program. TIME: starting from 1 day after surgery. DURATION: 3 days. NUMBER OF SESSIONS: 3 sessions. EXERCISE PROGRAM: leg press, knee extension, plantar flexion, hip abduction). In each session, the participant was asked to perform each exercise for the maximum number of repetitions until the intended intensity was reached. The exercise was evaluated until the elastic band at which the patient reported this intensity was found. PROGRAM INTENSITY: the point at which the patient reported perceived exertion (5-6) of 10 on the Borg CR-10 scale after 2 repetitions of each exercise on the first day as warm-up.	TYPE: as the intervention. TIME: as the intervention. DURATION: as the intervention. NUMBER OF SESSIONS: as the intervention. EXERCISE PROGRAM: ankle plantar/dorsal flexion (3 sets/20 repetitions), active knee extension with isometric contraction (8 sec/10 repetitions), isometric hip adductors/abductors contraction.	3 days	Western Ontario and McMaster Universities Osteoarthritis Index
Christiansen et al. (2015) [23]	Colorado, 2011-2012	RCT	NCT01333189	26	Type: postoperative rehabilitation (with weight-bearing biofeedback training plus control training). Duration: 6 weeks. Exercises: static bilateral stance, sit-to-stand, unilateral stance, lunging activities. Program intensity: progressively increasing difficulty, including weight shifting, depth, and speed based on patient tolerance and ability to perform tasks. Progression: unilateral stance activities added once patients can bear 100% body weight on the surgical limb; lunging activities introduced after achieving symmetrical weight bearing during sit-to-stand exercises.	Type: postoperative rehabilitation program (outpatient physical therapy and home-based exercises). Duration: 6 weeks. Type of exercises: passive and active ROM exercises, weight-bearing functional activities, stationary biking, modalities (ice and heat), and education. Daily over the 6-week period.	6.5	Lower limb weight-bearing ratios
Hamilton et al. (2020) [24]	UK, 2013	RCT	NCT01849445	334	Type: postoperative rehabilitation program. Duration: 6 weeks. Number of sessions: 18. Exercise types: range of motion, strengthening, proprioception, and walking gait.	Type: postoperative rehabilitation program. Duration: 6 weeks. Number of sessions: 18. Exercise types: self-directed home exercise-based protocol, which focused on unloaded bending of the knee to promote range of motion and using the weight of the limb to strengthen the quadriceps muscle (with a stationary knee).	13	Oxford knee score
Jakobsen et al. (2014) [26]	Denmark	RCT	NCT01351831	82	Type: postoperative rehabilitation program plus the control training. Duration: 7 weeks. Number of sessions: 14. Exercise types: progressive strength training for knee extension and leg press. Sets per session: 2 of each exercise. Rest between sets (in seconds): 60-180. Rest between repetitions (in seconds): 0. Contraction failure in each set: Yes. Rest between training sessions (in hours): 48-72. Anatomical definition of the exercise (exercise form): Yes. Repetition maximum: 8-12. Time under tension per exercise (in seconds): 128-192. Program intensity: The training loads were adjusted on a set-to-set basis to reflect the relative loads. If the patient initially was unable to lift the lightest weight in the knee extension machine, weight bands were used at the ankle.	Type: postoperative supervised physical rehabilitation without PST. Duration: 7 weeks. Number of sessions: 14. Exercise types: Warming up - Range of motion exercises - Stretching exercises - Functional training - Balance training	6	Maximal distance walked in 6 minutes
Lee et al. (2021) [28]	Korea	RCT	KCT00005129	38	Type: Postoperative progressive dynamic balance training (PDBT) plus the control training. Duration: 6 weeks. Timing: Initiated on the 3rd day after surgery. Exercise types: The program includes a dynamic balance training program (PDBT), which entails various activities. In the first two weeks, participants rotated their torsos while sitting on a chair, standing up, and lifting their heels while standing. In the following weeks, the activities progressed, including torso twisting while standing, walking sideways, using a step box to step up and down, marching, marching with turns, using a step box to step sideways, practicing tandem gait, walking around a cone, tiptoe gait, and changing directions while walking. Program intensity: Each session lasted 30 minutes with an inter-rest period of 30-40 seconds. There were two sets of 10 repetitions for each movement.	The control group had a postoperative inpatient rehabilitation program.		1. The Western Ontario and McMaster Universities (WOMAC) Osteoarthritis Index. 2. Pain pressure threshold. 3. Range of motion (ROM)
van Leeuwen et al. (2014) [29]	The Netherlands	RCT	NTR2278	22	Type: Postoperative rehabilitation program plus the control training. Duration: 6 weeks. Exercise types: - For the first training session (3 sets of 15 repetitions each): 1. Leg press. 2. Step up 1 leg. 3. Squat. 4. Leg extension 1 leg. During the first exercise (leg press), patients were asked to perform the maximum number of repetitions with the selected weight. For the second and third exercises, the intensity was increased by increasing the range of motion before using dumbbells. Both the uninvolved and the involved limbs were trained. The exercises were to be performed two to three times per week by the strength training group. If there was any pain or discomfort, the program was modified, but the intensity was to remain as high as possible. Program intensity: Training weights were adjusted according to the patients' abilities in relation to the number of repetitions. If more or less than 15 repetitions were performed, the weight for the next set was adjusted by approximately 3% per repetition.	The standard training group received information, advice, exercise for daily activities, walking with aids, mobility maintenance, and aerobic training (e.g., walking and cycling).		1. Isometric knee extensor strength. 2. Voluntary activation. 3. Chair stand. 4. 6-minute walk test (6MWT)
Liao et al. (2020) [31]	Taiwan, 2017-2019	RCT	ChiCTR--IPRIPR--17012106	60	Type: Postoperative rehabilitation program. Time: 1 month after surgery. Duration: 12 weeks. Type of exercises: The exercise regimen included resistance elastic training. The targeted exercises were seated chest press, seated row, seated shoulder press, hip circumduction, leg press, and leg curl. Participants in the exercise group (EG) performed 3 sets of 10 to 20 repetitions for each movement. The exercise loads were set according to the patients' perception of exertion on the 15-point Borg scale, ranging from "somewhat hard (13 grade rating)" to "hard (15 grade rating)." The program intensity involved progressive resistance load training, with adjustments made every 2 weeks based on the color of the Thera band.	Type: Standard care. Time: As the intervention. Duration: As the intervention. Types of exercises: Active and passive range of motion exercises, stretching exercises, and functional reconditioning exercises. These exercises were performed for both the operated and non-operated legs.	4	Functional performance: (1) Timed Up and Go (TUG), (2) gait speed, (3) forward reach, (4) single leg stance, (5) timed chair rise
Teissier et al. (2020) [35]	France	RCT	NR	20	Type: Postoperative rehabilitation (ECC-CON program). Duration: 4 weeks. Type of exercises: Eccentric exercises comprising 3–4 sets of 5–10 quadriceps eccentric flexions on the leg press, and 5–10 eccentric flexions of the hamstring muscles with seated leg curls and pulley. A rest period of approximately 5 minutes was allowed between sets. The concentric exercises are the same as the concentric group. Program intensity: The initial loading was based upon body weight percentage (25–30%), with regular increases during the program.	Type: Postoperative rehabilitation (CON program). Duration: As per intervention. Type of exercises: Participants were trained alternately for knee extensors and flexors. Each exercise consisted of 3–5 sets with 5–10 repetitions. A rest period of 3–5 minutes was provided between the sets. The program's intensity was based on a weight equivalent to 60–80% of each individual's estimated 1 repetition maximum (1 RM).	1	Performance-based physical function: (1) Timed up and go test, (2) 10-meter walk test, (3) isokinetic assessment.
Tilp et al. (2023) [36]	Austria	RCT	39/75/63ex 2020/21	24	Type: Bilateral postoperative rehabilitation. Duration: 3 weeks. Type of exercises: Strength training included 4 exercises (leg press, calf raise, knee flexion, knee extension) performed bilaterally on each side. Each exercise comprised 4 sets of 15 repetitions, with 1 minute of rest between sets. The program intensity was adjusted so that the patient could complete 15 repetitions in each set. As a result, the final 3 repetitions should be very exhausting.	As the intervention, but exercises were done unilaterally on the affected leg only.	0.75	Isometric knee extension strength

**Table 2:** Baseline characteristics. SD: standard deviation; No.: number; NR: not reported.

Study ID	Study groups	Age, mean (SD)	Sex (male), No. (%)	BMI, mean (SD)	Height, mean (SD)	Knee arthroplasty		Arthroplasty location	
						Unilateral, No. (%)	Bilateral, No. (%)	Left, No. (%)	Right, No. (%)
Bade et al. (2017) [22]	Intensive therapy (84)	63±8	39 (46%)	31±5	NR	84 (100%)	0 (0%)	NR	NR
	Conventional therapy (78)	64±7	34 (44%)	30±5	NR	78 (100%)	0 (0%)	NR	NR
Husby et al. (2018) [25]	Intensive therapy (21)	59.6±20.6	10. (47.6%)	31±5.3	NR	21 (100%)	0 (0%)	NR	NR
	Conventional therapy (20)	60.3±22.3	8 (40%)	28.6±5.1	NR	20 (100%)	0 (0%)	NR	NR
Jorgensen et al. (2017) [27]	Intensive therapy (31)	64.8±8.3	16 (51.6%)	29.8±4.8	NR	31 (100%)	0 (0%)	NR	NR
	Conventional therapy (24)	64.4±8.7	10 (41.6%)	28.4±2.8	NR	24 (100%)	0 (0%)	NR	NR
Lenssen et al. (2006) [30]	Intensive therapy (21)	70±8.5	6 (28.5%)	NR	NR	21 (100%)	0 (0%)	NR	NR
	Conventional therapy (22)	67±7	5 (22.7%)	NR	NR	22 (100%)	0 (0%)	NR	NR
Lowe et al. (2012) [32]	Intensive therapy (56)	67.8±8.4	24 (42.8%)	31.3±6.2	NR	56 (100%)	0 (0%)	31 (55.3%)	24 (42.8%)
	Conventional therapy (51)	70.7±9.45	21 (41.17%)	29.2±5.8	NR	51 (100%)	0 (0%)	22 (43.1%)	29 (56.8%)
Nakamura et al. (2020) [33]	Intensive therapy (24)	72.3±6.1	3 (12.5%)	25.8±3.4	1.53±7.8	24 (100%)	0 (0%)	NR	NR
	Conventional therapy (25)	73.9±6	4 (16%)	27.4±3.9	1.51±4.8	25 (100%)	0 (0%)	NR	NR
Núñez-Cortés et al. (2024) [34]	Intensive therapy (20)	70.6±6.9	10 (45%)	29.3±4.6	NR	20 (100%)	0 (0%)	NR	NR
	Conventional protocol (20)	71.6±7.2	12 (60%)	28.5±4.1	NR	20 (100%)	0 (0%)	NR	NR
Christiansen et al. (2015) [23]	Intensive therapy (13)	68.2±8.6	7 (53.8%)	83.5±18.2	1.7±0.1	13 (100%)	0 (0%)	NR	NR
	Conventional therapy(13)	66.6±8.1	6 (46.2%)	79.3±12.2	1.7±0.1	13 (100%)	0 (0%)	NR	NR
Hamilton et al. (2020) [24]	Intensive therapy (163)	66.8±9.46	66 (40.7%)	31.34±5.76	NR	NR	NR	NR	NR
	Conventional therapy (171)	68.2±9.44	63 (37.8)	31.50±6.18	NR	NR	NR	NR	NR
Jakobsen et al. (2014) [26]	Intensive therapy (35)	65±13.1	14 (40%)	94.0±19.8	1.7±0.01	35 (100%)	0 (0%)	NR	NR
	Conventional therapy (37)	62.6±8.4	16 (43%)	88.2±17.1	1.6±0.09	37 (100%)	0 (0%)	NR	NR
Lee et al. (2021) [28]	Intensive therapy (19)	72.05±5.15	0 (0%)	25.27±2.67	1.5±0.05	0 (0%)	19 (100%)	NR	NR
	Conventional therapy (19)	71.89±5.44	0 (0%)	26.75±4.03	1.5±0.05	0 (0%)	19 (100%)	NR	NR
Leeuwen et al. (2014) [29]	Intensive therapy (10)	71.8±7.5	7 (70%)	27.9±4.6	NR	NR	NR	NR	NR
	Conventional therapy (8)	69.5±7.1	4 (50%)	27.9±3.1	NR	NR	NR	NR	NR
Liao et al. (2020) [31]	Intensive therapy (30)	72.22±7.75	NR	28.54±3.88	NR	30 (100%)	0 (0%)	11 (37%)	19 (63%)
	Conventional therapy (30)	69.79±6.72	NR	27.25±4.36	NR	30 (100%)	0 (0%)	13 (43%)	17 (57%)
Teissier et al. (2020) [35]	Intensive therapy (10)	72.1±6.3	8 (40%)	31.1±6.1	NR	NR	NR	NR	NR
	Conventional therapy (10)				NR	NR	NR	NR	NR
Tilp et al. (2023) [36]	Intensive therapy (11)	59.1±6.1	4 (36.3%)	29.8±3.9	NR	11 (100%)	0 (0%)	NR	NR
	Conventional therapy (11)	62.5±9.7	6 (54.5%)	32.4±7.3	NR	11 (100%)	0( 0%)	NR	NR

Quality of the Included Studies

**Figure 2:**
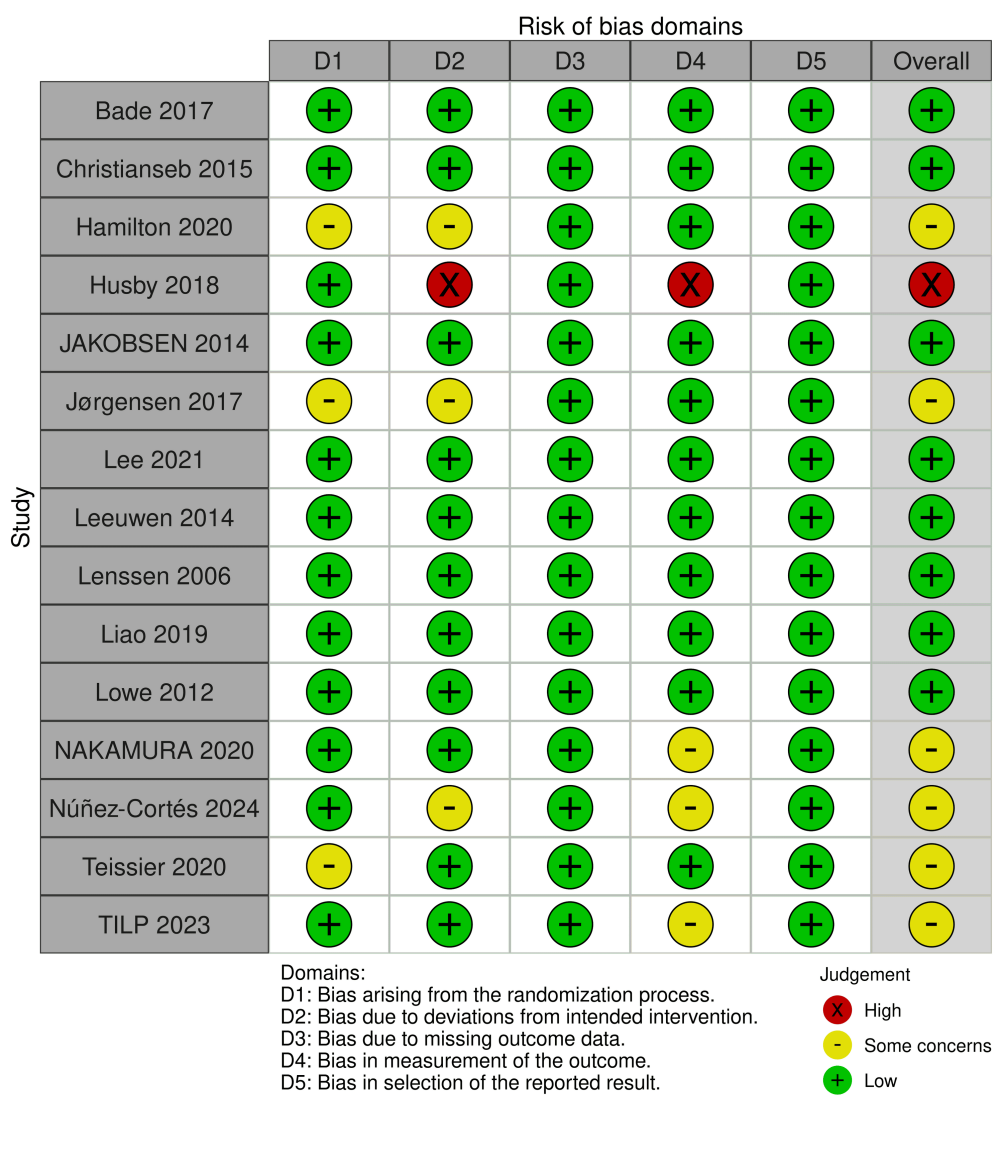
Risk of bias graph summary for randomized controlled trials (RCTs) made by the Risk of Bias 2 (ROB 2) tool. References: [22-36].

Regarding the ROB 2 tool, one study was found to be high in the possibility of bias, as two of their criteria were associated with a high risk of bias. On the other hand, eight studies were related to a low risk of bias, as all domains showed a low risk of bias. Finally, six studies had some concerns regarding the risk of bias. All details are shown in Figure 2.

Outcomes

Physical Measures

**Figure 3:**
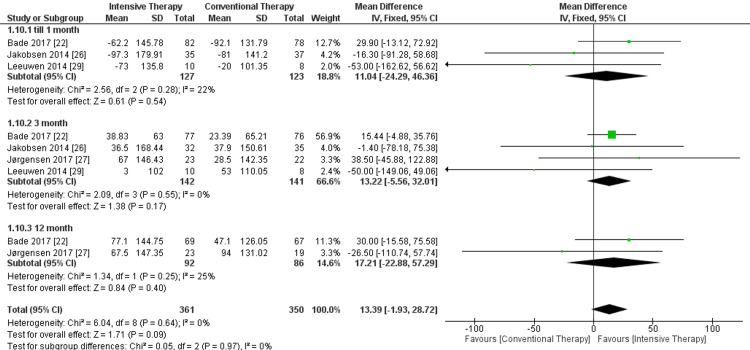
Forest plot of six or 10-minute walk test. Bade et al. [22], Jakobsen et al. [26], Jørgensen et al. [27], and Leeuwen et al. [29].

Six or 10-minute walk test: In the follow-up till one month, the ITT group showed no significant difference in the walking distance in meters when compared to the CTT group, and the pooled results were MD = 11.04, 95% CI = -24.29 to 46.36, and P-value = 0.54. Regarding the three-month follow-up, four studies were included, and the walking distance had no significant difference in ITT in comparison to CTT (MD = 13.22, 95% CI = -5.56 to 32.01, P-value = 0.17). When followed up for 12 months, only two studies were included, and there was no significance between the two comparator arms. Finally, the overall results showed a non-significant superiority for ITT in comparison to CTT regarding the outcome, and pooled results were MD = 13.39, 95% CI = -1.93 to 28.72, and P-value = 0.09. The overall results of the study were homogeneous, as P-value = 0.64 and I² = 0% (Figure 3).

**Figure 4:**
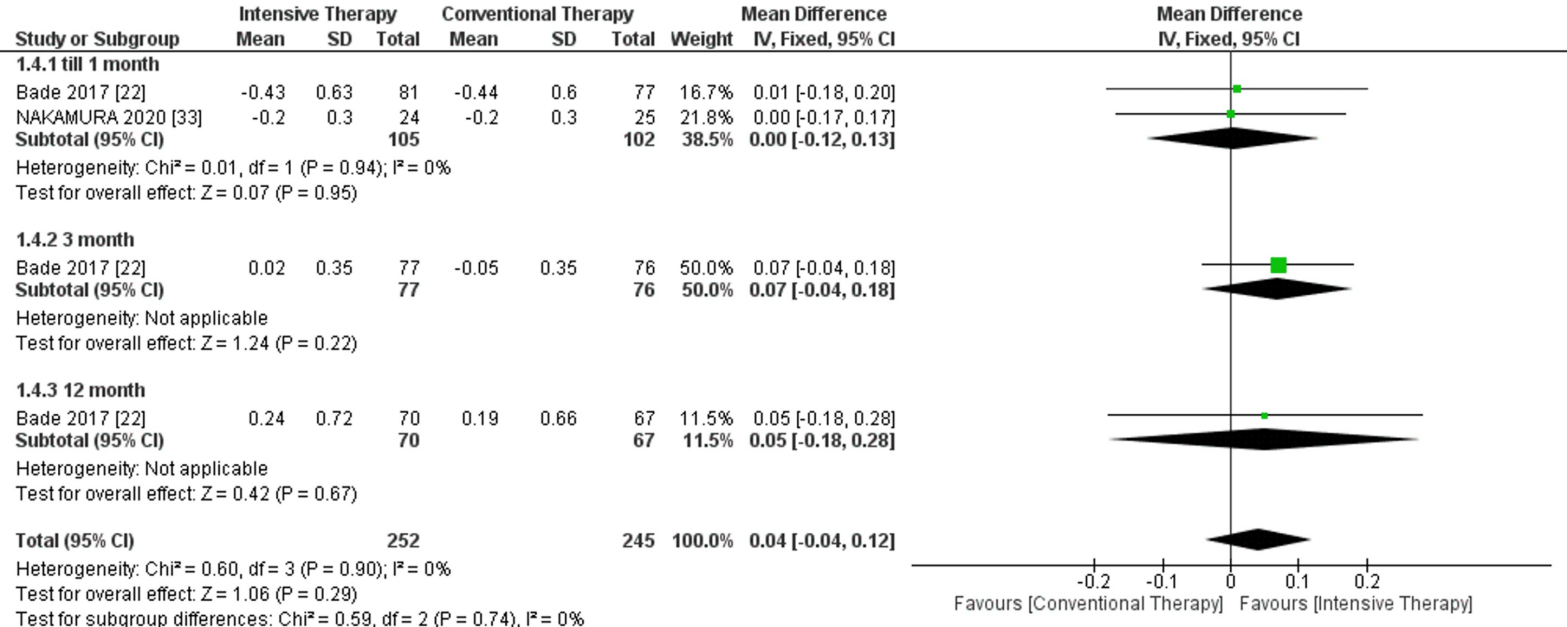
Forest plot of quadriceps strength. Bade et al. [22] and Nakamura et al. [33].

Quadriceps strength: In the follow-up until one month, two studies were included. The ITT showed no significant improvement in the quadriceps strength by kilogram (kg) (P-value = 0.95). Additionally, when followed up for three months, only one study was included. Same as the 12-month follow-up, and both showed no significance between the two comparator arms. The overall results demonstrated no superiority between ITT and CTT in terms of strength, and pooled results were MD = 0.04, 95% CI = -0.04 to 0.12, and P-value = 0.29. The study's overall results were homogeneous, as P-value = 0.29 and I² = 0% (Figure 4).

**Figure 5:**
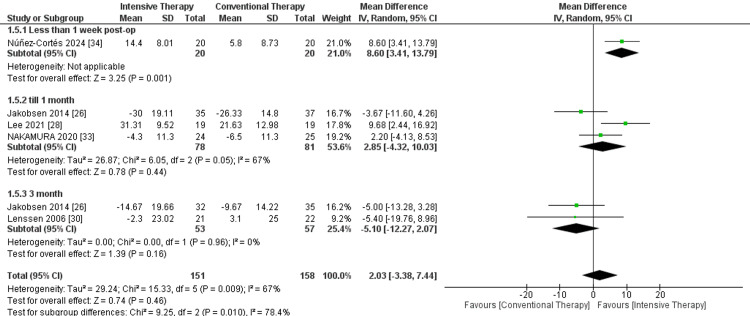
Forest plot of range of motion (ROM) flexion. Jakobsen et al. [26], Lee et al. [28], Lenssen et al. [30], Nakamura et al. [33], and Núñez-Cortés et al. [34].

ROM flexion: ​​​​In the follow-up of one week, only one study was included and showed that ITT resulted in significant improvement in ROM flexion (MD = 8.60, 95% CI = 3.41 to 13.79, P-value = 0.001). In the follow-up of one month and three months, the analyzed studies showed no significance between the two comparator arms. The overall result showed superiority for ITT in comparison to CTT regarding the outcome, although results were non-significant, and pooled results were MD = 2.03, 95% CI = -3.38 to 7.44, and P-value = 0.46 (Figure 5).

**Figure 6:**
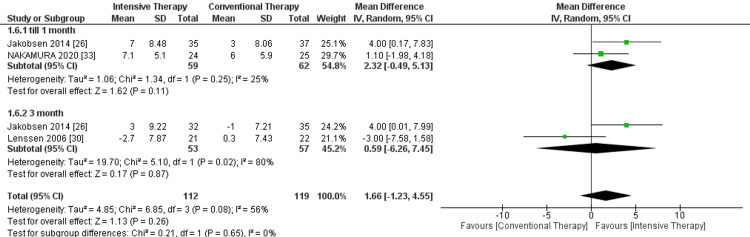
Forest plot of range of motion (ROM) extension. Jakobsen et al. [26], Lenssen et al. [30], and Nakamura et al. [33].

ROM extension: In the follow-up till one month, two studies were included. ITT showed no significant improvement in the ROM extension (MD = 2.32, 95% CI = -0.49 to 5.13, P-value = 0.11). Additionally, in the follow-up of three months, ITT showed no significant effect on the ROM extension therapy (MD = 0.59, 95% CI = -6.26 to 4.45, P-value = 0.87). The overall results showed no significant effect of ITT in comparison to CTT regarding the outcome, and pooled results were MD = 1.66, 95% CI = -1.23 to 4.55, and P-value = 0.26 (Figure 6).

**Figure 7:**
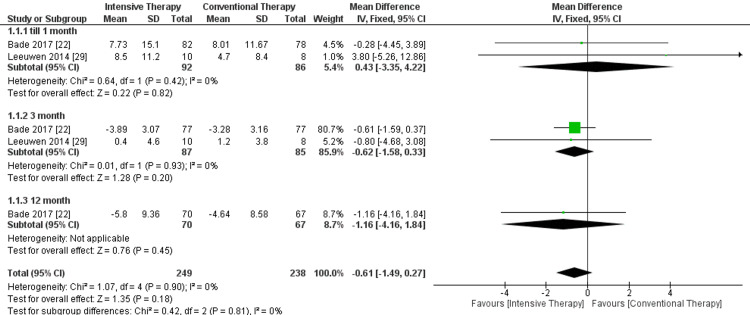
Forest plot of stair test. Bade et al. [22] and Leeuwen et al. [29].

Stair test (seconds): No significant difference was found at all follow-up durations. The overall results of the analyzed studies showed no significance in the reduction of time, favoring the highly intensive exercise therapy in comparison to CTT, and pooled results were MD = -0.61, 95% CI = -1.49 to -0.27, and P-value = 0.18. The study's overall results were homogeneous, as P-value = 0.90 and I² = 0% (Figure 7).

**Figure 8:**
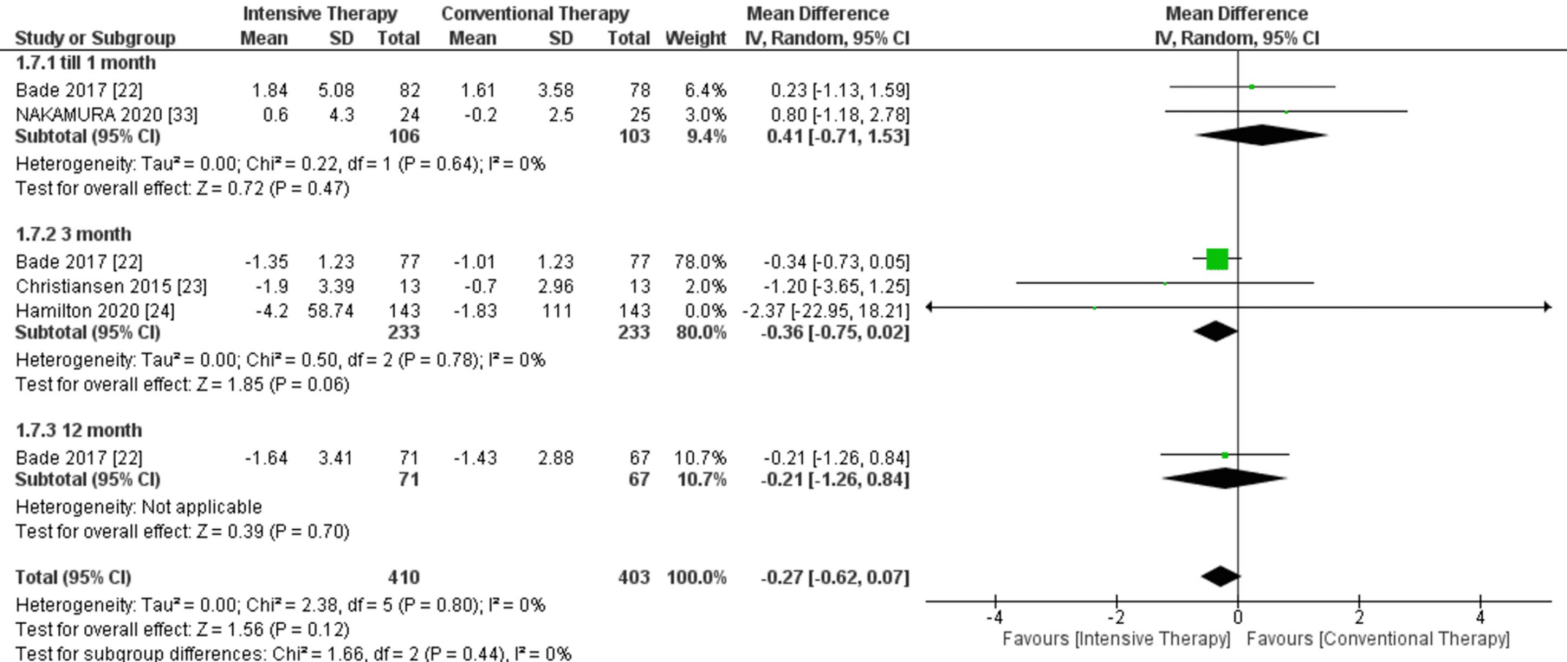
Forest plot of time up and go. Bade et al. [22], Christiansen et al. [23], Hamilton et al. [24], and Nakamura et al. [33].

Timed up and go: There was no significance between the high-intensity training compared to low-intensity training at any follow-up duration. The overall results of the analyzed studies also showed no significance of highly intensive exercise therapy in comparison to CTT, and pooled results were MD = -0.27, 95% CI = -0.62 to 0.07, and P-value = 0.12. The study's overall results were homogeneous, as P-value = 0.80 and I² = 0% (Figure 8).

Questionnaires

**Figure 9:**
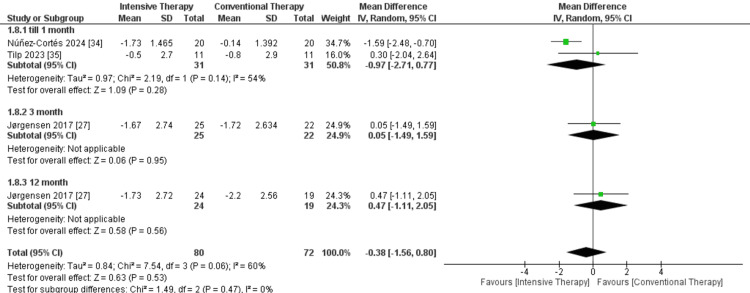
Forest plot of visual analog scale (VAS) score. Jørgensen et al. [27], Núñez-Cortés et al. [34], and Teissier et al. [35].

VAS score: In the follow-up till one month, two studies were included, and the result showed a non-significant decrease in pain in ITT on VAS when compared to CTT, with MD = -0.97, 95% CI = -2.71 to 0.77, and P-value = 0.28. Moreover, in the follow-up of three months and 12 months, there were no significant differences between the two comparators. The overall results demonstrated no significance between the two comparators, and pooled results were MD = -0.38, 95% CI = -1.56 to 0.8, and P-value = 0.53. The overall results of the study were heterogeneous, as P-value = 0.06 and I² = 60% (Figure 9).

**Figure 10:**
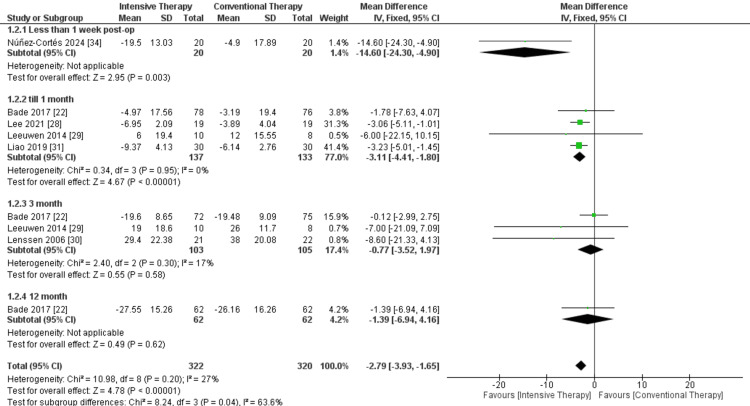
Forest plot of Western Ontario and McMaster Universities Osteoarthritis Index (WOMAC) scores. Bade et al. [22], Lee et al. [28], Leeuwen et al. [29], Lenssen et al. [30], Liao et al. [31], and Núñez-Cortés et al. [34].

WOMAC score: In the less than one-week follow-up duration, only one study was included and showed that ITT resulted in significant improvement in WOMAC (MD = -14.60, 95% CI = -24.30 to -4.90, P-value = 0.003). Regarding the follow-up till one month, the results were also significant as ITT showed better improvement compared to CTT (MD = -3.11, 95% CI = -4.41, -1.80, P-value <0.0001), and the results were homogenous (P = 0.95; I² = 0%). On the other hand, at three and 12 months follow-up, there was no significance between the two comparators. The overall results of the analyzed studies showed the significance of ITT in WOMAC when compared to CTT, and pooled results were MD = -6.26, 95% CI = -10.53 to 1.98, and P-value 0.004. The overall results of the study were heterogeneous, as P-value > 0.00001 and I² = 87% (Figure 10).

Discussion

In TKA rehabilitation, ITT has demonstrated significant benefits over CTT in the functional outcome of ROM flexion in the follow-up of less than one week. ITT consistently shows a non-significant difference in walking distances in six and 10-minute walk tests at all follow-up durations, indicating no improvement in mobility and endurance. No significant improvements in quadriceps strength were noted with ITT, whether in short-term follow-ups or in long-term follow-ups. ITT did not significantly affect ROM extension at both included follow-up durations. Overall, the stair test favored ITT without a significant difference, as ITT showed no significance in reducing the completion time of the stair test. The outcomes related to the questionnaire showed that pain, which is measured with the VAS score, showed no significant decrease regarding any follow-up duration. The WOMAC score improved significantly with ITT early on and at three months, taking into consideration that the less than one week period included only one study.

Choosing the right intensity and duration of training is crucial for enhancing muscle strength, as most previous studies had intensity ranging from low to moderate [37]. Rooks et al. discovered that a preoperative progressive resistance program can enhance postoperative functional performance and muscle strength, though they found no significant improvements in patient-reported outcomes [38]. Another randomized controlled study indicated that preoperative high-intensity strength training reduces pain, enhances lower limb muscle strength, and improves functional task performance [39].

The ability to sit, stand up from a chair, and walk are key activities of daily living and indicators of mobility and functional capacity. However, several studies have found that preoperative training does not improve these activities with CTT associated with low intensity [29,40,41]. In our study, we found no difference between the interventions regarding the time up and go between the ITT and CTT in the postoperative training, which indicates that sitting and standing from the chair are affected by whether the intervention was ITT or CTT and whether the training is done before or after the surgery.

Sun et al. [3] added balance training to high-intensity training, which in the end resulted in the enhancement of the knee flexor-extensor strength and balance of patients with end-stage knee OA in the short term and helped in improving early outcomes after OA. Furthermore, improved balance makes early rehabilitation easier and reduces fall risk due to impaired proprioception [3]. Additionally, Husby et al. [25], who investigated the effect of maximal strength training in comparison to the standard rehabilitation, added balance training to the main intervention and obtained results showing significance in leg press and knee extension muscle strength in short-term follow-up. These previous results, when adding balance training, should be considered in rehabilitation programs due to the possible positive outcome found by this addition.

Pain is recognized as one of the primary symptoms in patients with OA. Thorstensson et al. found that OA patients often feared that exercise would further damage their knee joints, and their concern is related to pain sensation [42]. Those who had never exercised believed it might harm the osteoarthritic joint. This study suggests that preoperative training could alleviate this fear, help patients manage pain, and encourage them to maintain exercise as part of a new lifestyle after surgery [42]. This finding could indicate that it is important to add preoperative exercise to the postoperative period to avoid the psychological effects.

One of the main factors that should be considered, resulting in the lack of effectiveness of strength training after TKA, is arthrogenic muscular inhibition (AMI) [24]. The AMI condition is characterized by a reduced neural drive to the quadriceps muscle, preventing its full activation [25]. AMI is linked to knee pain, swelling, inflammation, and structural damage, all common post-TKA issues. This inhibition likely prevented patients from reaching the intensity required to stimulate muscle hypertrophy and improve functional performance [43]. AMI is a protective reflex response to joint damage, which inhibits muscle activation to prevent further harm but consequently limits the effectiveness of strength training, whether in high or low intensity [43]. Jakobsen et al. found that including leg extension and leg press exercises in a rehabilitation program did not improve outcomes compared to a program that omitted these exercises [26]. Jakobsen et al. linked their finding to AMI. Our findings also showed slight improvement regarding isometric knee flexion and extension, which may be due to AMI [44].

The included studies mainly focused on postoperative exercise, as it plays a vital role in restoring joint function. Early and consistent movement prevents stiffness and ensures the knee joint functions properly [28,34,45]. Exercise helps in managing pain. It stimulates the production of endorphins, which are the body’s natural painkillers, and are expected to lower the pain, and according to our findings, the reduction of pain would not have a difference whether the follow-up is long or short [1].

We had lower heterogeneity in comparison to the previous meta-analysis. We exclusively included randomized clinical trials and avoided observational study designs to improve the quality of our findings. We conducted a subgroup analysis to enhance the evidence and make the results more comprehensive, resulting in sufficient evidence that we could rely on. Our study included all the extractable outcomes that were mentioned in the included studies. Our study also had several limitations, as we introduced all types of physical therapy without specification, except that they were all postoperative exercises. The intensity of the exercise training varied among the included studies; some studies extended the training period, others increased the number of sessions, and others increased the intensity of the exercise, which may result in possible heterogeneity among the included population. The types of exercise were not similar among all the included studies, with some studies combining different types of training, such as endurance and balance training. We recommend that future researchers focus on covering all possible outcomes to give more accurate results and have the same inclusion criteria. We recommend conducting more standardized and specific studies that clearly define and categorize the types of physiotherapy interventions, including detailed protocols for strength training, range of motion exercises, functional training, and any combined modalities. Future investigations should establish standardized exercise intensity parameters, including specific duration, frequency, and progression criteria.

## Conclusions

In TKA rehabilitation, ITT shows early benefits in improving ROM flexion within the first postoperative week. ITT also showed better WOMAC score improvement in the early follow-up. However, ITT does not significantly outperform CTT in walking distances, quadriceps strength, ROM extension, or pain reduction across various follow-up periods. Overall, ITT offers slight early benefits with no long-term advantage over CTT. It may be recommended to add preoperative training to the postoperative exercise. We suggest that there is no need for high-intensity exercises, as the yield would be similar to conventional methods, suggesting that the additional effort and resources required for intensive protocols may not be justified, given the similar therapeutic yields achieved with standard conventional methods. However, some outcomes are not covered across various studies, which requires more future research focusing on both early and long-term outcomes.
